# Quasi‑Φ_0_‑Periodic Supercurrent at Quantum Hall Transitions

**DOI:** 10.1021/acsnano.5c05294

**Published:** 2025-07-24

**Authors:** Ivan Villani, Matteo Carrega, Alessandro Crippa, Elia Strambini, Francesco Giazotto, Vaidotas Mišeikis, Camilla Coletti, Fabio Beltram, Kenji Watanabe, Takashi Taniguchi, Stefan Heun, Sergio Pezzini

**Affiliations:** † NEST, Istituto Nanoscienze-CNR and Scuola Normale Superiore, Piazza San Silvestro 12, Pisa 56127, Italy; ‡ CNR-SPIN, Via Dodecaneso 33, Genova 16146, Italy; § Center for Nanotechnology Innovation, Laboratorio NEST, Istituto Italiano di Tecnologia, Piazza San Silvestro 12, Pisa 56127, Italy; ∥ Research Center for Electronic and Optical Materials, National Institute for Materials Science, 1-1 Namiki, Tsukuba 305-0044, Japan; ⊥ Research Center for Materials Nanoarchitectonics, National Institute for Materials Science, 1-1 Namiki, Tsukuba 305-0044, Japan

**Keywords:** graphene, Josephson junction, quantum Hall, supercurrent, quantum devices

## Abstract

The combination of superconductivity and quantum Hall (QH) effect is regarded as a key milestone in advancing topological quantum computation in solid-state systems. Recent quantum interference studies suggest that QH edge states can effectively mediate a supercurrent across high-quality graphene weak links. In this work we report the observation of a supercurrent associated with transitions between adjacent QH plateaus, where transport paths develop within the compressible two-dimensional bulk. We employ a back-gated graphene Josephson junction, comprising high-mobility CVD-grown graphene encapsulated in hexagonal Boron Nitride (hBN) and contacted by Nb leads. Superconducting pockets are detected persisting beyond the QH onset, up to 2.4 T, hence approaching the upper critical field of the Nb contacts. We observe an approximate Φ_0_ = *h*/2*e* periodicity of the QH-supercurrent as a function of the magnetic field, indicating superconducting interference in a proximitized percolative phase. These results provide a promising experimental platform to investigate the transport regime of percolative supercurrents, leveraging the flexibility of van der Waals devices.

## Graphical abstract

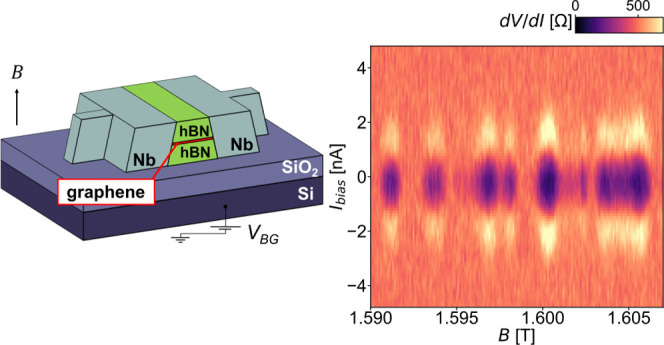

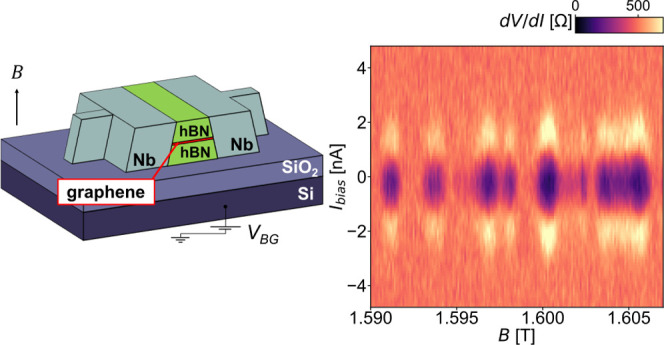

Quantum computing hardware resilient to environmental disturbances can be engineered based on specific topological phases of matter, which allow nonlocal information storage and manipulation via quasiparticle exchange in real space.^1,2^ This paradigm can be realized in systems where QH states are interfaced with s-wave superconductors,^3^ supporting non-Abelian anyons such as Majorana zero modes^4^ and parafermions.^5,6^ These platforms are regarded as fundamental building blocks for topological quantum computation, paving the way toward a universal set of quantum gates.^7,8^ Consequently, significant efforts have been dedicated to overcome the intrinsic challenges of proximitizing QH states, both in III-V semiconductors^9–14^ and graphene.^15–23^ hBN-encapsulated graphene devices are considered an ideal experimental platform, owing to their ability to support highly transparent one-dimensional contacts^24,25^ which facilitate Andreev reflection both in the integer^16^ and fractional^19^ QH regime. Additionally, these devices enable coherent edge-state propagation over micrometers,^26,27^ further enhancing their suitability for quantum transport experiments. The first observation of a supercurrent (SC) in the QH regime by Amet et al.^15^ was made possible by Josephson junctions with this configuration. The SC was attributed to the coupling between chiral Andreev Edge States (CAES), hybrid electron-hole modes propagating along the graphene-superconductor interface,^17^ and QH edge states, forming a closed loop. The resulting chiral supercurrent, subject to Aharonov-Bohm interference, is expected to display a 2Φ_0_ = *h*/*e* periodicity as a function of the applied magnetic field,^28,29^ implying that it oscillates at half the frequency of a conventional nonchiral Josephson current. However, an unexpected Φ_0_ = *h*/2*e* periodicity was reported in ref 15 for a SC in the QH regime. Subsequent studies^30,31^ suggested that counterpropagating channels capable of independently carrying a SC emerge due to charge accumulation at etched graphene edges,^32^ effectively forming a superconducting quantum interference device (SQUID). Recent calculations show that the Φ_0_-periodicity could also arise from finite coupling between the CAES wave functions in the short junction limit.^33^ By miniaturizing device edges (width *W* and length *L* < 330 nm) to enhance phase coherence,^34^ Vignaud et al. were able to observe a 2Φ_0_-periodic chiral QH-SC at filling factor ν = 2.^20^ More recently, Barrier et al.^21^ reported a QH-SC carried by one-dimensional domain walls within the bulk of minimally twisted bilayer graphene (hence not relying on physical edges) persisting up to magnetic fields very close to the critical field of the superconducting contacts. Altogether, these results highlight the crucial influence of sample boundaries, including both graphene-superconductor and graphene-vacuum interfaces, in QH-SC coupling. However, charge transport in the QH regime can also involve bulk extended states at transitions between different QH plateaus.^35^ In such cases, two-dimensional systems undergo a localization-delocalization transition,^36^ which was modeled (semiclassically) in terms of percolation^37^ through a network of QH droplets generated by long-range potential fluctuations.^38^

In this work we present experimental evidence of QH-SC in Nb-contacted encapsulated graphene, persisting up to *B* = 2.4 T, close to the Nb upper critical field (*B*_c_ = 3.2 T). The SC is observed at QH plateau-plateau transitions and exhibits an approximate Φ_0_ periodicity as a function of magnetic field. Our findings suggest a mechanism analogous to low-field Fraunhofer pattern in planar Josephson junctions, enabled by the formation of percolative bulk channels. The interference involves different areas throughout the device, governed by the interplay of applied electromagnetic fields and doping near the graphene-superconductor interface.

## Results and Discussion

### Device Properties at Zero and Low Magnetic Field

We experimentally investigate the QH-SC coexistence using a back-gated monolayer graphene Josephson junction, whose structure is schematically depicted in Figure 1a. The Josephson junction has length *L* = 400 nm and width *W* = 3 μm (in line with the device dimensions in ref 15); an optical microscopy image of the device is shown in Figure 1b together with a sketch of the measurement configuration. All measurements are performed in a Leiden Cryogenics dilution refrigerator equipped with cryogenic filtering, at a base temperature of 40 mK (except otherwise indicated). We employ high-mobility graphene single crystals grown by chemical vapor deposition (CVD)^39–42^ and encapsulated in hBN flakes via the dry van der Waals pickup method.^24,42,43^ Top and bottom hBN flakes are each 30 nm thick, resulting in a total stack thickness *t* = 60 nm. High-transparency graphene-Nb interfaces are obtained by combining dry etching of hBN^24^ with DC magnetron sputtering of 60 nm thick Nb contacts^44^ (further details on the fabrication process are reported in the Methods section). Nb maintains its superconducting state at fields well exceeding the QH onset for high-mobility graphene (e.g., a QH onset as low as 50 mT was measured in Hall bar devices based on the same graphene crystals in ref 42). The device is fabricated on a Si/SiO_2_ substrate (285 nm thick SiO_2_), and the underlying p-doped Si functions as a back-gate (BG).

**Figure 1:**
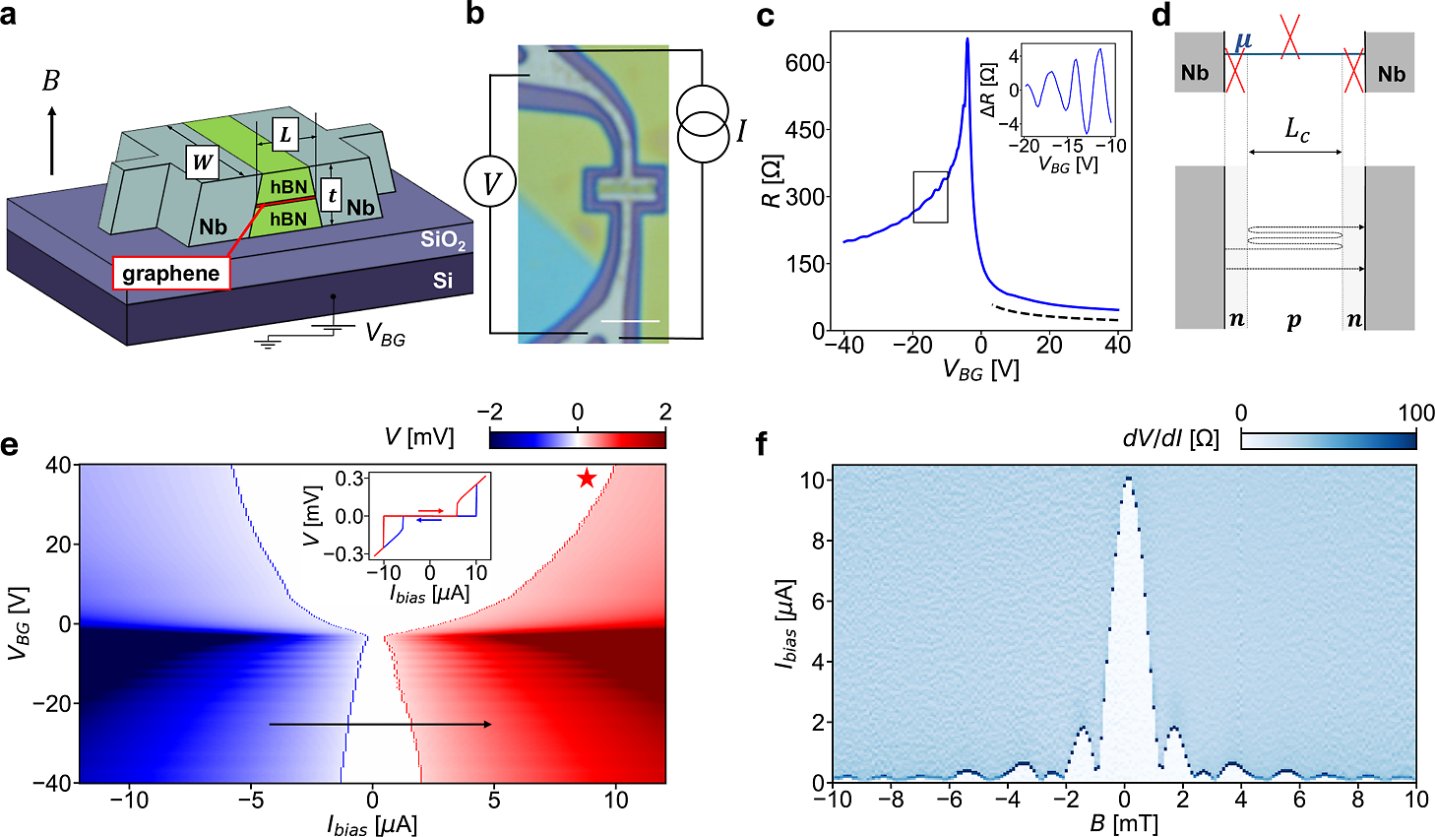
(a) 3D schematics of the device layout and gating configuration. When applied, the magnetic field *B* is orthogonal to the substrate plane. Device dimensions: *L* = 400 nm, *W* = 3 μm, *t* = 60 nm (schematics is not to scale). (b) Optical microscopy image of the junction. Scalebar: 3 μm. The four-probe measurement layout is indicated: a current *I* (combining DC and AC components as specified in the main text) is applied to the junction, and the voltage drop *V* is measured. (c) Representative backgate sweep: sample resistance R = *V*/*I* as a function of the backgate voltage *V*_BG_. *T* = 4.2 K, *B* = 0 T. The normal state resistance is measured by applying a sufficiently large current bias and by measuring the voltage drop *V* (*Inset*) Fabry-Pérot oscillations as a function of *V*_BG_ over the range highlighted by the rectangle in the main panel. A polynomial background is removed as discussed in the Supporting Information, Section S1.1. (d) *Upper panel* Schematics of the doping across the device in the p-type (hole) doping regime: Dirac cones in red are traced according to the local doping (μ is the chemical potential). The n-type (electron) doping induced by Nb leads to the formation of a n-p-n cavity. *Lower panel* Sketch of ballistic transport across the device. Charge carriers have a finite probability to be reflected at the p-n interfaces, leading to Fabry-Pérot interference analogous to an optical cavity. (e) Voltage drop *V* as a function of DC current bias *I*_bias_ and backgate voltage *V*_BG_. The current sweep direction is indicated by the black arrow. The dotted blue and red lines correspond respectively to the retrapping and switching currents. *T* = 40 mK, *B* = 0 T (*Inset*) *V*-*I*_bias_ curve for *V*_BG_ = 40 V showing switching-retrapping behavior. Arrows indicate the sweep direction. (f) Differential resistance d*V*/d*I* (obtained by numerical differentiation of the measured DC voltage drop with respect to the applied DC current bias) as a function of the out-of-plane magnetic field *B* (in the ±10 mT range) and current bias *I*_bias_ at *V*_BG_ = 40 V, displaying a Fraunhofer interference pattern. Darker points, indicating a peak in d*V*/d*I*, correspond to the transition from the dissipationless to the dissipative regime. *T* = 40 mK.

When measuring the normal-state resistance of the device as a function of the BG voltage (*V*_BG_) we obtain asymmetric curves, as shown in Figure 1c, with the charge neutrality point (CNP) shifted to slightly negative *V*_BG_ (−3.6 V in this specific sample). This shift is attributed to Fermi level pinning at the Nb contacts, that results in n-type (electron) doping in the neighboring graphene region, as shown in Figure 1d. For positive *V*_BG_ the resistance *R* approaches the Sharvin limit^44^ (dashed black line in Figure 1c), indicating high interface transparency (up to Tr ∼ 0.8 estimated following ref 44). For *V*_BG_ < *V*_CNP_, a n-p-n cavity forms across the junction,^25^ leading to a larger resistance on the p-type (hole) doping side. This stems from the p-n interfaces acting as partially reflecting barriers (as exemplified in Figure 1d), with an estimated transparency Tr ∼ 0.3 at *V*_BG_ = – 40 V. In this regime, Fabry-Pérot (FP) oscillations are observed (see inset to Figure 1c). These oscillations are a clear signature of ballistic transport as they can occur only if electrons maintain coherence while traveling across the device.^25,44,45^ In ballistic n-p-n cavities, the resistance oscillates as a function of the Fermi wavevector *k*_F_, with minima satisfying the resonance condition *k*_F_ *L*_c_ = *m*π + π/2 (where *L*_c_ is the cavity length and *m* the cavity mode number).^25^ From the periodicity of the oscillations in the p-type doping regime we estimate the cavity length to be ∼200 nm. A comparable cavity length value is estimated also in the n-type doping regime, where a n-n′-n cavity forms and FP oscillations are observed. The oscillations are, however, much less visible owing to the higher interface transparency (further details are provided in Supporting Information, Section S1.1).

Figure 1e shows a colormap of the voltage drop *V* across the junction, measured while sweeping the DC current bias (*I*_bias_) at different *V*_BG_ values in the (−40, 40) V range. The DC bias sweep direction is indicated by the black arrow. Supercurrent is measured in the white central region corresponding to zero voltage drop. The critical current (*I*_c_, identified as the boundary between the dissipationless and dissipative regions) is modulated by *V*_BG_ and approaches values up to 10 μA at large n-type doping (red star in Figure 1e). This value and the corresponding supercurrent density *J*_s_ ≃ 3.3 μAμm^–1^ are in line with graphene Josephson junctions with similar length, as shown in refs 15 and 44 , demonstrating comparable sample quality. A minimum of *I*_c_ is observed at the CNP, while on the p-doping side *I*_c_ is consistently smaller than on the corresponding n-doping side, due to the reduced transparency at the p-n interfaces. FP oscillations are visible also in the supercurrent as a modulation of *I*_c_: in this case, maxima in the supercurrent correspond to minima in the resistance (see details in Supporting Information, Section S1.2). The device exhibits a pronounced switching-retrapping behavior (as can be seen also from the *V*-*I*_bias_ curve in the inset in Figure 1e). This behavior is known to originate from electron heating in the dissipative branch.^46–48^ Additional characterization of the device at zero magnetic field, which includes a discussion about multiple Andreev reflections and the *I*_c_ × *R*_n_ figure of merit is reported in Section S2 of the Supporting Information.

In the low magnetic field limit, for |*B*| < 10 mT, a Fraunhofer interference pattern (Figure 1f) is observed. Deviations from a perfectly regular pattern can be attributed to slight inhomogeneities in the supercurrent distribution in the direction perpendicular to the current flow.^49^ Additionally, in the Meissner phase at low magnetic fields, flux focusing is known to increase the effective flux through the junction,^50^ leading to a smaller periodicity than expected.^44,51,52^ Following the argument in ref 15, based on the geometry of our device we expect a focusing factor ≃1.8 (defined as the ratio between the effective field in the junction and the applied field *B*). This value is in good agreement with ≃1.7, estimated as the ratio between the expected theoretical position of the first Fraunhofer minima and their observed position (for further details see the Supporting Information, Section S3).

As the magnetic field increases, the amplitude of the SC does not follow the expected suppression based on the standard Fraunhofer dependence. Instead, regions with supercurrent persist. These regions, known as “superconducting pockets”,^44^ are characterized by a partial or complete suppression of the differential resistance and can survive up to large magnetic fields reaching the Tesla range. In the semiclassical regime, below the onset of QH states, where *r*_c_ > *L*/2 (*r*_c_ = ℏ*k*_F_/*eB* is the cyclotron radius), we observe superconducting pockets not only in the n-type doping regime, as reported in ref 44, but also for p-type doping. Additional discussion regarding the SC pockets in the semiclassical regime is reported in the Supporting Information, Section S4. SC pockets are observed in the QH regime, as well, as discussed in the following.

### Supercurrent up to 2.4 T

The plot in Figure 2a shows the differential resistance d*V*/d*I*, measured with a lock-in amplifier as a function of BG voltage *V*_BG_ and magnetic field *B*, while applying a relatively large AC current of 100 nA on top of a 200 nA DC bias. Linecuts of d*V*/d*I* at fixed *B* = 0.5 T and *B* = 2.5 T are reported in Figure 2b. On the n-type doping side, to the right of the CNP, a regular Landau fan diagram spreads out from the CNP, while the pattern on the p-type side is heavily influenced by the p-n interfaces discussed above, with FP oscillations merging with Landau levels, and following a *B* ^2^ dispersion with respect to *k*_F_, as discussed in the Supporting Information, Section S1.2. The observation of FP oscillations at finite magnetic field provides further evidence of the ballistic nature of electrical transport. Given the wide aspect ratio of the junction (*L*/*W* = 7.5), d*V*/d*I* shows an oscillating behavior with maxima corresponding to the position of QH plateaus^53,54^ and reduced differential resistance within them (as can be also seen in the linecut at *B* = 2.5 T shown in Figure 2b).

**Figure 2:**
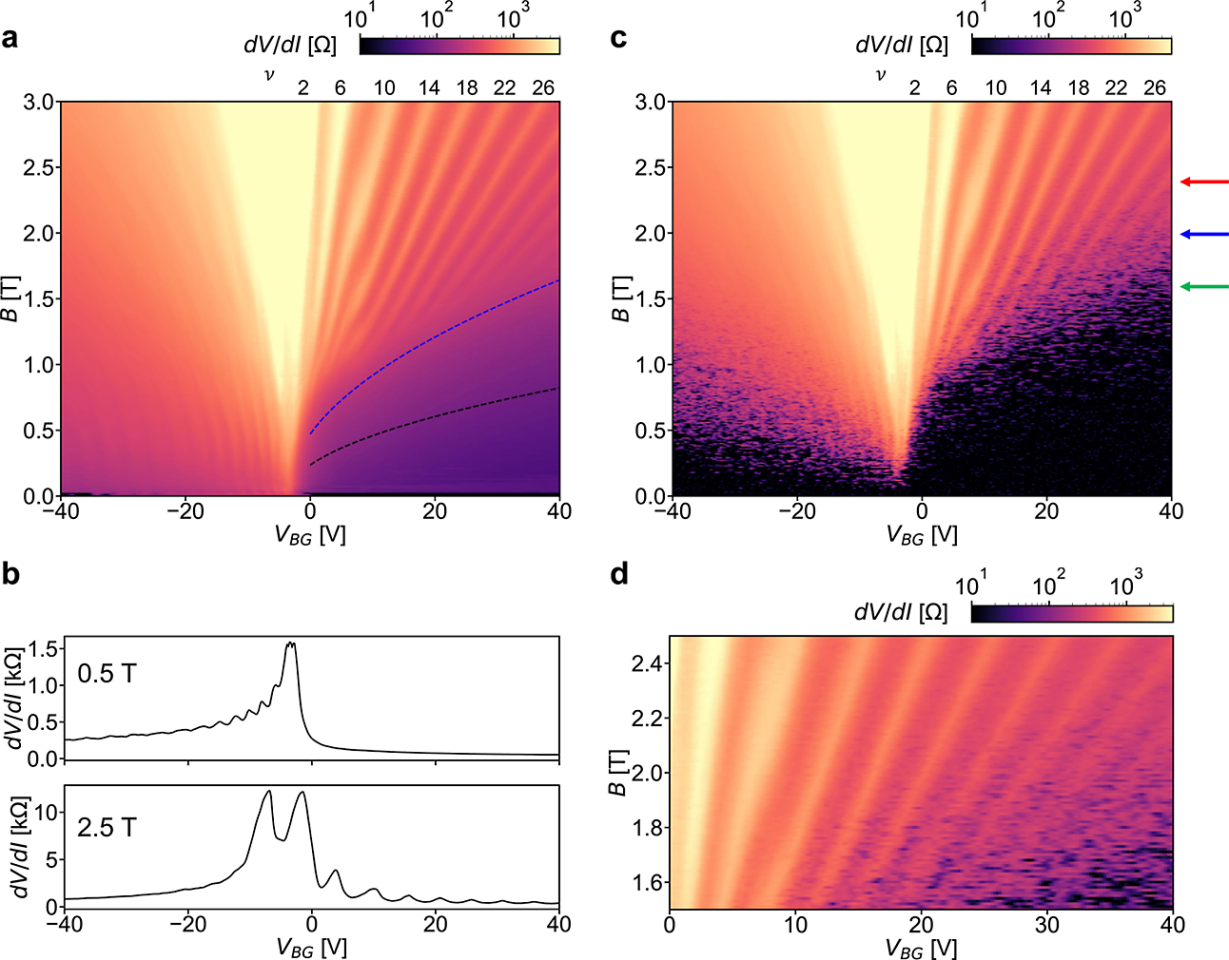
(a) Landau fan diagram (d*V*/d*I* as a function of *B* and *V*_BG_). The differential resistance d*V*/d*I* is measured with a lock-in amplifier, by applying a 100 nA AC bias excitation on top of a fixed 200 nA DC bias excitation. The dashed black line represents the expected QH-semiclassical regime crossover calculated from the geometrical dimension *L* = 400 nm of the device, while the blue dashed line is obtained by considering the estimated FP cavity length *L*_c_ = 200 nm, as explained in the main text. The two lines are obtained from the equation ℏ*k*_F_/*eB* = *L*/2, respectively for *L* = 400 nm and *L* = 200 nm. The Fermi wavevector $k_{F}=\sqrt{\pi n}$ is calculated from the gate-dependent carrier density *n* = *f*(*V*_BG_ – *V*_CNP_) (*f* is the gate lever arm). (b) Linecuts from panel a of d*V*/d*I* vs *V*_BG_ at fixed magnetic field. At *B* = 0.5 T, on the p-type doping side, FP oscillations are still observed, as they evolve with magnetic field. At *B* = 2.5 T, d*V*/d*I* shows a nonmonotonic behavior, as discussed in the text. (c) Landau fan diagram obtained by measuring the differential resistance d*V*/d*I* with a lock-in amplifier by applying a small (500 pA) AC bias and no DC bias. QH superconducting pockets appear as dark spots on top of d*V*/d*I* oscillations, absent in panel a. *T* = 40 mK. The colored arrows indicate the position of the acquisitions shown in Figure 3. (d) Zoom of the region in c where SC pockets are seen in the QH regime, appearing as darker spots indicating locally suppressed d*V*/d*I*.

At large magnetic field values, in the n-type doping regime (*V*_BG_ > *V*_CNP_), the condition *r*_c_ = *L*/2 marks the crossover between the semiclassical and the QH regime. In our device, however, the boundary appears to be determined by a length scale shorter than the junction length, which we identify as the FP cavity length. This is shown in Figure 2a where the separation line corresponding to the junction geometrical length, *L* = 400 nm (black dashed line) clearly does not match the semiclassical to QH regime separation. Instead, considering the FP cavity length (blue dashed line, corresponding to 200 nm), a better agreement is obtained.

In the data shown in Figure 2a the applied current bias (comprising a 100 nA AC excitation on top of a DC 200 nA excitation) is large enough to suppress any trace of supercurrent. By lowering the AC bias down to 500 pA and setting the DC bias to zero, numerous superconducting states are observed, not only in the semiclassical regime, but also in the QH regime, as shown in Figure 2c. In Figure 2c the SC pockets can be identified as dark spots on top of the Landau fan diagram, absent in the large bias acquisition of Figure 2a. They correspond to a partial local suppression of the differential resistance and can be appreciated by directly comparing Figure 2c with Figure 2a. A zoom of the region of the Landau fan diagram where SC pockets are observed is reported in Figure 2d.

To further substantiate these observations of QH-SC, we perform measurements of d*V*/d*I* with AC excitation of 100–200 pA, as a function of *V*_BG_ and *I*_bias_, for selected values of magnetic field. These acquisitions are reported in Figure 3. Figure 3a shows data for *B* = 1.6 T (corresponding to the green arrow in Figure 2c). Some pockets are observed in the QH regime, starting at ν > 14, with *I*_c_ in the 1 nA range (similar to refs 15 and 20 ). For *V*_BG_ ≳ 25–30 V, which brings the system close to the QH onset, pockets with larger *I*_c_ are also observed. At *B* = 2 T (Figure 3b, blue arrow in Figure 2c) multiple pockets are observed at relatively large filling factor. These pockets are mainly located between d*V*/d*I* peaks, i.e., in the transition region between contiguous QH plateaus. The lower panel in Figure 3b compares the line cuts taken at *I*_bias_ = 0 nA and *I*_bias_ = 4 nA (the latter large enough to suppress the supercurrent). Several SC states, marked with “*”, can be seen as suppression of d*V*/d*I* in the red line as compared to the black one. At *B* = 2.4 T (Figure 3c, red arrow in Figure 2c), residual supercurrent regions are present for ν > 26 (see arrow in Figure 3c). The amplitude of the pockets is rapidly suppressed with increasing temperature, and no supercurrent is observed for *T* > 200 mK (see Supporting Information, Section S5 for details).

**Figure 3:**
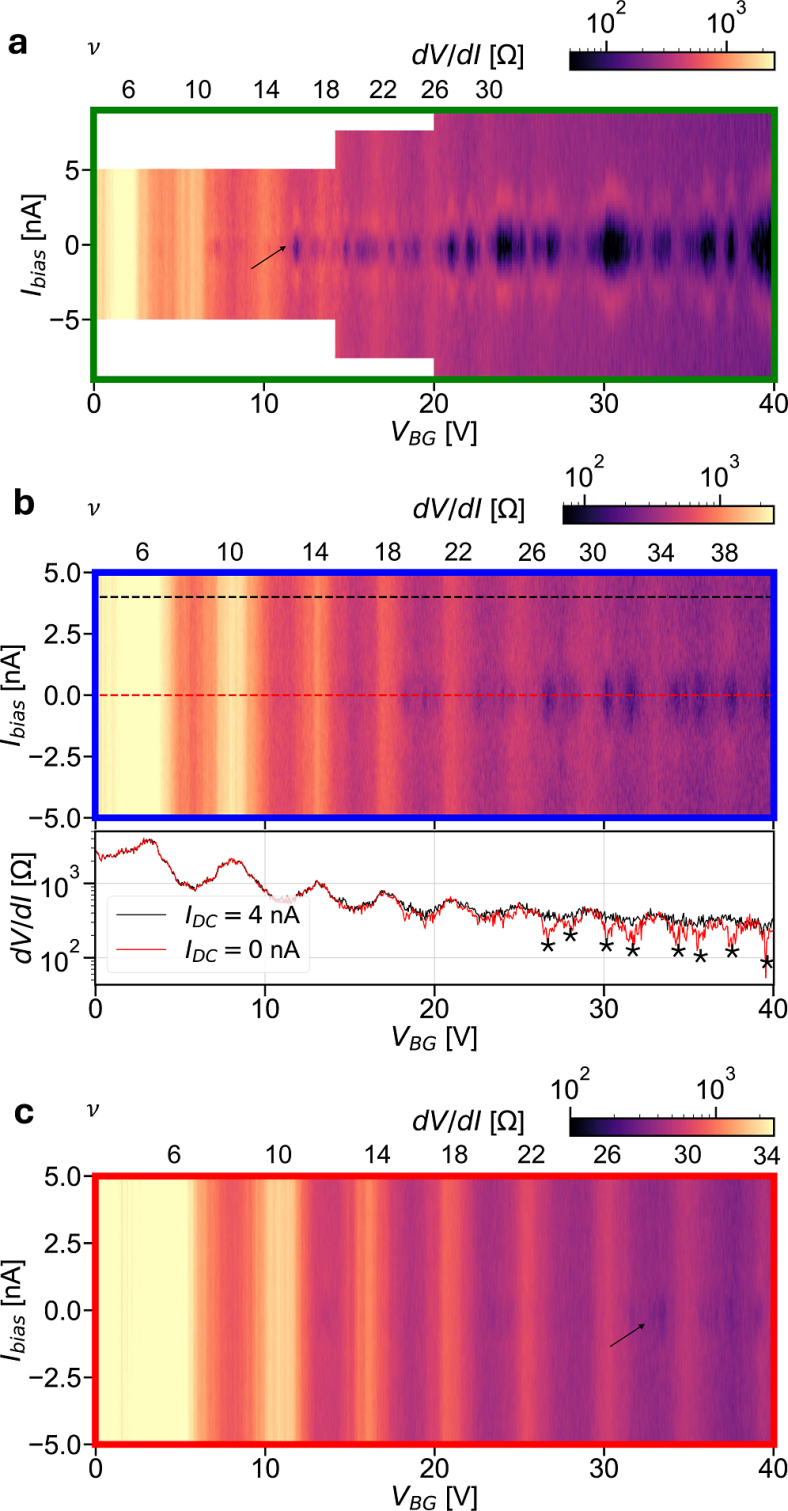
Differential resistance d*V*/d*I* as a function of backgate voltage *V*_BG_ and applied DC bias *I*_bias_ for selected values of the magnetic field *B*. d*V*/d*I* was measured with a lock-in amplifier by applying a small AC bias excitation, as indicated in the following. (a) Acquisition at *B* = 1.6 T, corresponding to the green arrow in Figure 2c. AC modulation: 100 pA in the (0, 14) V range, 150 pA in the (14,20) V range, 200 pA in the (20, 40) V range. Black arrow indicates the chosen pocket for the acquisitions shown in Figure 4. (b) Acquisition at *B* = 2.0 T, corresponding to the blue arrow in Figure 2c. AC bias: 100 pA. Linecuts at *I*_DC_ = 0 nA (red line) and *I*_DC_ = 4 nA (black line), are shown in the lower panel. Superconducting pockets, indicated by * appear as a partial suppression of d*V*/d*I* in the zero DC bias line compared to the finite DC bias case. (c) Acquisition at *B* = 2.4 T, corresponding to the red arrow in Figure 2c. AC bias: 100 pA. A weak suppression of d*V*/d*I* at large filling factor can be seen (indicated by the black arrow).

### Periodicity of Supercurrent at QH Transitions

Previous reports of supercurrent in the QH regime showed a clear periodicity of the SC pockets with magnetic field. For junctions with a large aspect ratio similar to our device,^15,30,31^ a Φ_0_ = *h*/2*e* periodicity was observed as a function of magnetic field, for fixed backgate voltage. In those cases, the SC was located on top of QH plateaus, hence it was carried exclusively by channels along the sample edges. In our experiment, instead, the suppression of d*V*/d*I* is not observed on the QH plateaus but in the transition regions between the plateaus. Although signatures of a SC at QH transitions are visible in data presented in refs 20 and 30 , a specific investigation of this phenomenon is currently lacking.

To elucidate these differences, we perform two dedicated measurements, shown in Figure 4: one at fixed *V*_BG_ (Figure 4a) and one at fixed filling factor ν (Figure 4c). The acquisitions are performed around *B* = 1.6 T in a 50 mT-wide magnetic field range, around the pocket indicated by the arrow in Figure 3a. In the first acquisition, shown in Figure 4a, *V*_BG_ = 11.6 V is kept constant and the magnetic field is varied, causing a change of the filling factor from ν = 15.8 to ν = 16.3. In the fixed-ν acquisition, shown in Figure 4c, magnetic field and backgate voltage are varied simultaneously to keep ν = 16.04 ± 0.02 (in the transition region between the plateaus at ν = 14.0 and ν = 18.0). No sharp periodicities emerge in both Figure 4a,c. However, an approximate periodicity can be appreciated from bunches of pockets that appear evenly spaced (see the guiding dashed lines).

**Figure 4:**
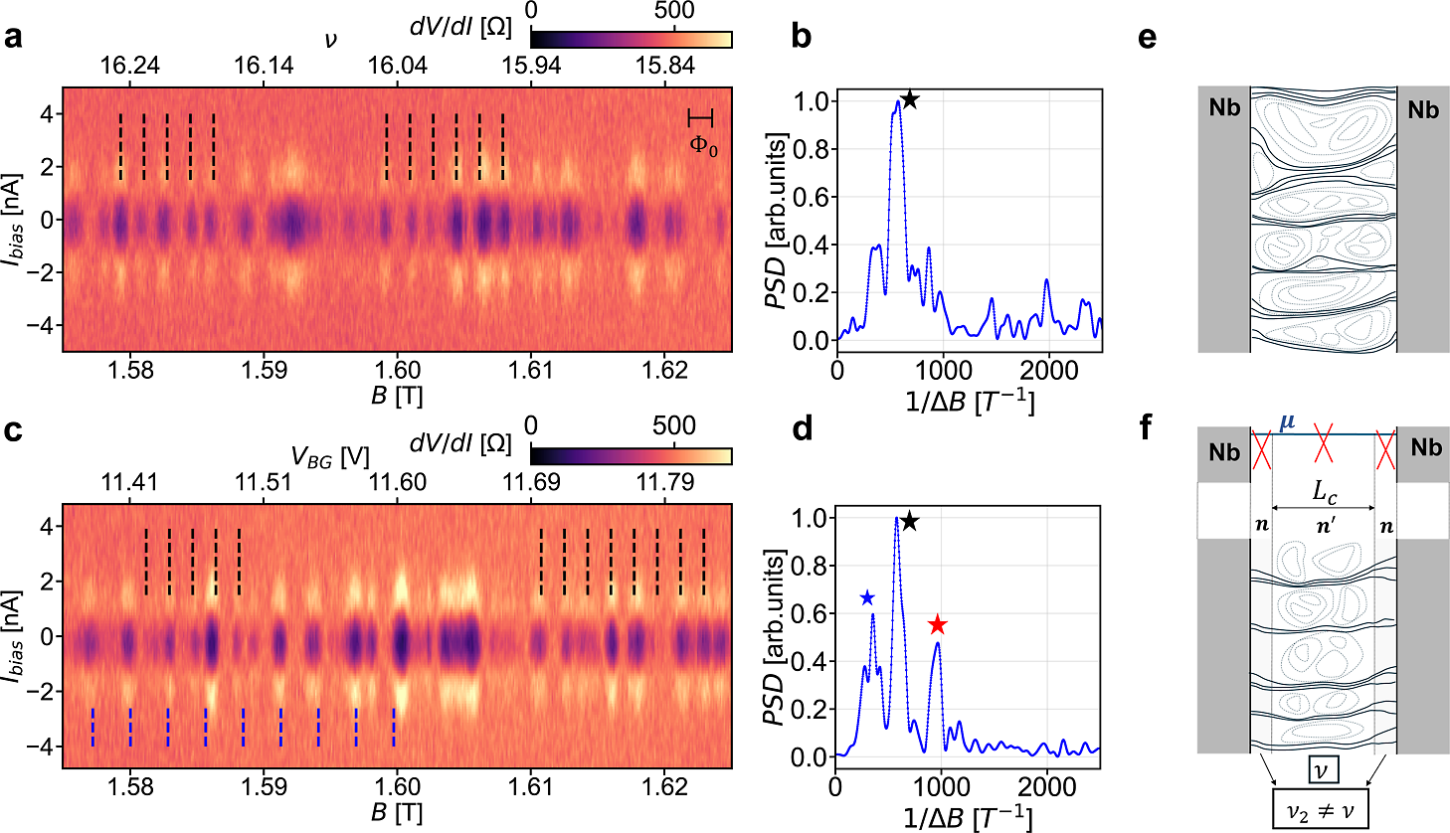
(a) Differential resistance d*V*/d*I* as a function of magnetic field *B* and DC bias current *I*_bias_, acquired at fixed *V*_BG_ = 11.6 V. d*V*/d*I* was measured with a lock-in amplifier by applying a 100 pA AC bias excitation on top of the sweeping DC bias. Black dashed lines correspond to Φ_0_-periodic oscillations on the full junction’s area (main peak in the Fourier spectrum in panel b). (b) Fourier transform (power spectral density) of zero-bias d*V*/d*I* data in panel a. (c) Same as panel a, acquired at fixed filling factor ν = 16.04 ± 0.02. Black dashed lines correspond to Φ_0_-periodic oscillations on the full junction’s area (main peak in the Fourier spectrum in panel d, indicated by the black star), while blue dashed lines correspond to Φ_0_-periodic oscillations on the smaller area corresponding to the FP cavity (peak in the Fourier spectrum in panel d indicated by the blue star). (d) Same as panel b, for data set in c. (e) Schematics of the transport mechanism in the percolation regime: random transport channels arise in the bulk via merging of QH droplets. (f) Same as panel e, considering the highly n-doped regions near the Nb contacts.

To further investigate these observations we perform a Fourier analysis on the d*V*/d*I* signal at zero DC bias. The resulting Fourier spectra are shown in Figure 4b (relative to Figure 4a) and Figure 4d (relative to Figure 4c). In the fixed-*V*_BG_ acquisition of Figure 4a, a main peak (black star in Figure 4b) appears at 1/Δ*B* = 575 T^–1^: this value corresponds to a period of Δ*B* ≃ 1.7 mT, which in turn corresponds to a flux variation of ΔΦ = Δ*B* × *L* × *W* ≃Φ_0_, where *L* = 400 nm and *W* = 3 μm are the physical dimensions of the device (note that, for *B* > *B*_c,1_ ≃ 180 mT,^55^ magnetic field penetrates the contacts, hence flux focusing does not play a role anymore). Dashed black lines in Figure 4a are traced according to this periodicity. We observe that superconducting pockets cluster into approximately equally spaced groups, interspersed with regions where their distribution appears more random. In the following we propose a mechanism which could explain the observed quasi-periodicity.

As previously mentioned, the observed superconducting pockets are located in the transition region between QH plateaus, where electrical currents percolate across merging QH droplets, as exemplified in Figure 4e. Near zero magnetic field, where interference patterns such as the one in Figure 1f are measured, multiple parallel transport channels are available throughout the entire device. The magnetic flux through the junction determines the interference between these channels,^51^ leading to the observation of maxima and minima as a function of magnetic field (i.e., the Fraunhofer pattern). In the percolative QH regime, exemplified in Figure 4e, the supercurrent density can spatially arrange in a way similar to the near-zero magnetic field state, i.e., it can distribute across the whole 2D bulk, though nonuniformly. As a result, superconducting interference as a function of the magnetic field leads to the observed Φ_0_-periodicity reported in Figure 4a,b. Since the shape and distribution of QH droplets evolves as the filling factor is varied (Figure 4a,b), the distribution of transport channels also changes, as discussed above. This evolution is driven by the details of the potential fluctuations across the graphene sheet, which likely account for the observed irregular quasi-periodicity. The available transport channels, forming randomly in the percolation regime within the bulk of the junction, are dephased with respect to each other. Therefore, one expects the supercurrent to be of the order of that of a single channel *I* ∼ *ev*_F_/*L*. Indeed, all measured pockets in the QH regime show a characteristic critical current of ∼1 nA, consistent with previous reports of QH-SC,^15,20^ and in good agreement with the expected value ∼*ev*_F_/*L*, taking into account the reduced Fermi velocity of QH channels^26,27^ in proximity to superconductors.^20^

In principle, the rearrangement of the QH droplets should be prevented by keeping the filling factor value fixed, but no sharp Φ_0_-periodicity is observed even in the constant-ν data (Figure 4c,d). On the contrary, as for the fixed-*V*_BG_ acquisition of Figure 4a,b, a main peak in the Fourier transform appears (black star in Figure 4d), corresponding to an approximate Φ_0_-periodicity. A second smaller peak develops at lower frequency (blue star in Figure 4d). We interpret this peak as evidence of Φ_0_-periodic oscillations stemming from a smaller area, whose dimensions correspond to the FP cavity previously discussed. As pointed out earlier, a n-n′-n cavity forms in the n-type doping regime, as sketched in Figure 4f. In the central area of the junction (*n*′) the filling factor value is kept constant by acting on *B* and *V*_BG_ in the constant-ν measurement configuration. However, the areas near the contacts host a different charge density that is determined by the interplay of Fermi-level pinning at Nb contacts and applied *V*_BG_. While at large magnetic fields FP interference is no longer observed due to cyclotron motion, the doping variation across the sample is preserved as it is determined by the effect of the Nb contacts. As a result, the local filling factor in the region close to the Nb contacts (ν_2_) varies as the magnetic field and *V*_BG_ are changed. Consequently, the QH droplets continue to evolve in these regions, leading to an uncontrolled rearrangement of the available transport channels. The extracted frequency for the second peak in Figure 4d, identified by the blue star and centered at 355 T^–1^, corresponds to an area of width *W* = 3 μm and length of 245 ± 30 nm. Dashed blue lines in Figure 4c are traced according to this periodicity. This length value is comparable to the estimated FP cavity length (∼200 nm), which also appears to determine the semiclassical-to-QH regime separation. Altogether, these observations indicate an interplay between the cavity originated by doping variations and the spatial distribution of SC-carrying percolative paths. A third peak (red star in Figure 4d) is also observed in the fixed-ν acquisition, located at a frequency value ascribable to the sum of the main oscillatory components. Such peak arises due to a coupling of the two identified periodicities, implying a net modulation dominated by the smaller supercurrent (further details are discussed in the Supporting Information, Section S6).

Percolative superconductivity is a phenomenon that is observed in various solid-state systems. It plays a key role in the behavior of quantum materials such as cuprates^56^ and oxide interfaces,^57^ but also granular superconductors^58^ of high technological relevance.^59^ In our experiment, we realize a synthetic percolative superconductor obtained via Josephson effect at QH transitions, which could serve as testbed for studying this transport regime, with the added flexibility provided by van der Waals devices. For example, we note that the scaling behavior of QH transitions in graphene appears to depend on the employed substrate (such as SiO_2_,^60^ hBN,^61^ hBN-on-graphite^62^). This suggests that substrate engineering could offer a means of tuning the induced SC.

## Conclusions and Outlook

In conclusion, we have demonstrated induction of superconducting states in the QH regime in Nb-contacted graphene Josephson junctions. We observe quasi-*B*-periodic SC oscillations, with a main peak in the Fourier spectrum at Φ_0_. Since superconducting pockets are present in the QH percolative regime, our findings point to an interference mechanism analogous to the low-field Fraunhofer pattern. Additionally, a second peak at lower frequency is detected when the filling factor in the central area of the sample is kept constant, indicating that interference takes place also over a smaller area, corresponding to the FP cavity, which is influenced by local doping at the Nb-graphene interfaces. Our findings provide evidence for a distinct mechanism for SC-QH coexistence, differing from edge-mediated transport^15^ and 2Φ_0_-periodic chiral supercurrent,^20^ with possible general implications for the investigation of percolative superconductors.

## Methods: Device Fabrication

We assemble the hBN-graphene-hBN stack using standard dry pick-up methods.^24,43^ We employ a poly­(bisphenol A carbonate) (PC) film deposited onto polydimethylsiloxane (PDMS)^43^ to combine micromechanically exfoliated hBN flakes and CVD-grown graphene as described in ref 42. After the assembly, we select target areas for device fabrication based on Raman spectroscopy signatures indicating minimal nanoscale strain variations.^42,63,64^ A poly­(methyl methacrylate) (PMMA) mask is used for electron beam litography (EBL) patterning. A first EBL step defines self-aligned one-dimensional superconducting contacts.^44^ A 15 s mild oxygen plasma step (10 W power) is performed prior to etching to ensure complete removal of polymer residues in the exposed areas. A mixture of CF_4_ and O_2_ (flow rate 20 and 2 sccm respectively) is used to etch through the entire 60 nm-thick stack in approximately 30 s at 25 W power. An additional 10 s mild oxygen plasma step is performed after CF_4_-O_2_, yielding an increased Nb-graphene interface transparency (by typically 20–30%). 60 nm-thick Nb contacts are then deposited using DC magnetron sputtering at a rate of ∼1 nm/s, with no adhesion layer deposited prior to Nb. Lift-off is performed in warm acetone (*T* = 50 °C) for 20 min. A second EBL patterning is used to define the device mesa, followed by the same RIE process used for the contacts. The sample is eventually cleaned in acetone at room temperature overnight.

## Supplementary Material



## Notes

Additional device characterization measurements: Fabry-Pérot oscillations (Figures S1, S2, S3, S4), multiple Andreev reflection and *I*_c_ × *R*_n_ product (Figure S5), flux focusing (Figure S6); superconducting pockets in the semiclassical regime (Figure S7); temperature dependence of pocket amplitude in the QH regime (Figures S8, S9); coupling of B-modulated supercurrents and sum frequency in the FFT spectrum (Figures S10, S11, S12, S13) (PDF)
