# Supplementary material for: Quasi‑Φ0‑Periodic Supercurrent at Quantum Hall Transitions
Source: ACS Nano. 2025 Jul 24;19(30):27370–8. doi: 10.1021/acsnano.5c05294 (PMC12333403; doi:10.1021/acsnano.5c05294)
Supplement: Supplementary file 1 [file nn5c05294_si_001.pdf]

Supporting Information to: "Quasi-Φ_0-periodic supercurrent at quantum Hall transitions"

Ivan Villani,[*,†] Matteo Carrega,[‡] Alessandro Crippa,[†] Elia Strambini,[†] Francesco Giazotto,[†] Vaidotas Mišeikis,[¶] Camilla Coletti,[¶] Fabio Beltram,[†] Kenji Watanabe,[§] Takashi Taniguchi,[‖] Stefan Heun,[*,†] and Sergio Pezzini[*,†]

†*NEST, Istituto Nanoscienze-CNR and Scuola Normale Superiore, Piazza San Silvestro 12, 56127 Pisa, Italy*

‡*CNR-SPIN, Via Dodecaneso 33, 16146 Genova, Italy*

¶*Center for Nanotechnology Innovation, Laboratorio NEST, Istituto Italiano di Tecnologia, Piazza San Silvestro 12, 56127 Pisa, Italy*

§*Research Center for Electronic and Optical Materials, National Institute for Materials Science, 1-1 Namiki, Tsukuba 305-0044, Japan*

‖*Research Center for Materials Nanoarchitectonics, National Institute for Materials Science, 1-1 Namiki, Tsukuba 305-0044, Japan*

E-mail: ivan.villani@sns.it; stefan.heun@nano.cnr.it; sergio.pezzini@nano.cnr.it

S1 Fabry-Pérot oscillations

S1.1 Estimating the FP cavity length from FP oscillations

In ballistic Josephson junctions, FP oscillations are observed as modulation of the resistance. Given the resonance condition $k_F L_c = m\pi + \pi/2$, the cavity length can be estimated from the period of resistance oscillations as a function of k_F, obtained by means of a Fourier

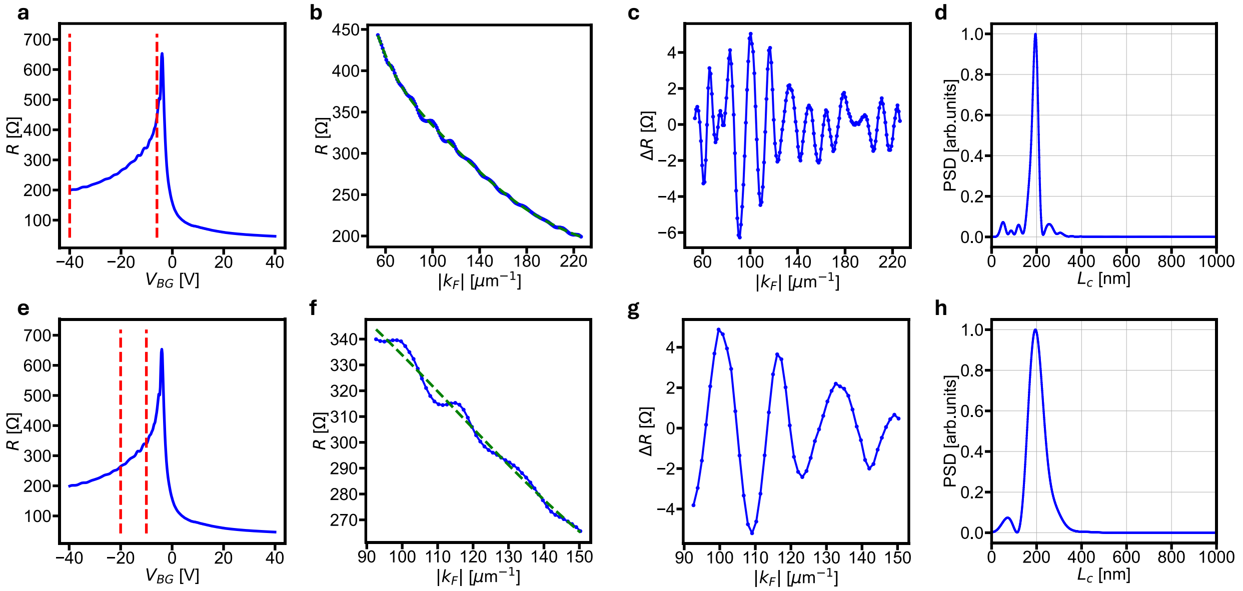

Figure S1: Analysis on FP oscillations in the p-type doping regime. (**a**) Normal state resistance as a function of V_{BG}. Vertical red dashed lines indicate the selected interval. (**b**) Normal state resistance R as funtion of Fermi wavevector k_F in the selected interval. The fitted polynomial background is shown as green dashed line. (**c**) R *versus* k_F signal, detrended of the background (here and in the main text labelled as ΔR). (**d**) Fourier transform (power spectral density) of data in panel **c**. Peak corresponds to $L_c \sim 200$ nm. (**e-h**) Same as **a-d**, but in a 10 V wide window ($V_{BG} = (-10, -20)$ V).

transform. An example is shown in Fig. S1, where we report the FP cavity length calculation in the p-type doping regime. The normal state resistance R values are plotted *versus* Fermi wavevector k_F in **b**. The Fermi wavevector $k_F = \sqrt{\pi n}$ is calculated from the gate-dependent carrier density $n = f(V_{BG} - V_{CNP})$ (f is the gate lever arm). Due to the requirement of an evenly spaced x-array to apply the Fourier transform, an interpolation procedure is applied to redefine the R *versus* k_F dataset such that k_F array is evenly spaced. To isolate FP oscillations from the background, a polynomial function (green dashed line in **b**) is fitted and then subtracted: resulting data are shown in Fig. S1c (the same is done in the inset of Fig. 1c in the main text). The Fourier transform is computed by using Welch's method (implemented through the `scipy.signal.welch` Python function). The result is shown in Fig. S1d: from the peak position we extract a cavity length of $\sim$ 200 nm. By performing the same procedure on narrower intervals of V_{BG} we obtain comparable results, as shown in the

example for the $V_{BG} = (-10, -20)$ V interval, Fig. S1e-h (peak corresponds to $\sim$ 200 nm).

In the n-type doping regime FP oscillations, originating in the n-n'-n cavity, have a much lower visibility due to the larger interface transparency (this limits their observability to a few V-wide window to the right of the CNP). FP oscillations in the $(0, 10)$ V and $(5, 15)$ V intervals are shown in Figs. S2a,c (plotted *versus* k_F), the corresponding power spectral densities in Figs. S2b,d. Peak in **b** corresponds to $L_c \sim 180$ nm while peak in **d** to $L_c \sim 220$ nm. Therefore, a cavity length of the order of $\sim$ 200 nm is estimated also in the n-type doping regime.

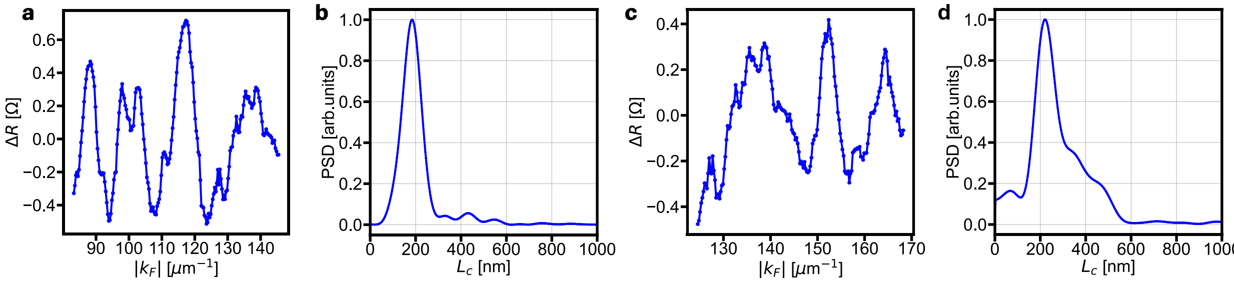

Figure S2: (**a**) Detrended FP oscillations in the $V_{BG} = (0, 10)$ V range, plotted as a function of Fermi wavevector. (**b**) Fourier transform (power spectral density) of data in panel **a**. Peak corresponds to $\sim$ 180 nm. (**c**) Detrended FP oscillations in the $V_{BG} = (5, 15)$ V range, plotted as a function of Fermi wavevector. (**d**) Fourier transform (power spectral density) of data in panel **c**. Peak corresponds to $\sim$ 220 nm.

S1.2 FP oscillations in the supercurrent and dispersion with magnetic field

At zero magnetic field, FP oscillations are observed not only as modulation of the normal state resistance, but also as modulation of the switching and retrapping currents.[1] This is shown in Fig. S3, where the voltage drop across the junction is plotted as a function of the sweeping DC bias at different V_{BG} values in the interval $(0, -15)$ V. The resistance outside of the supercurrent branch ($I_{bias} = -6$ μA) is shown in the right plot. Local maxima in the supercurrent are aligned to minima in the resistance, as highlighted by the dashed guiding black lines.

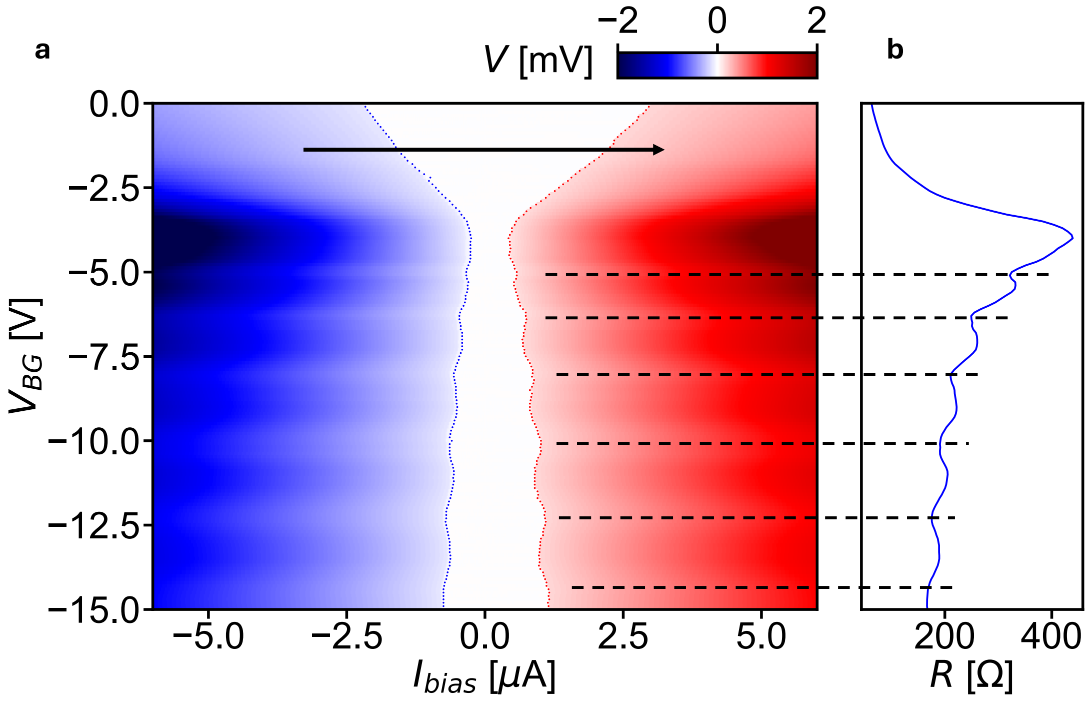

Figure S3: (**a**) Voltage drop as a function of DC current bias I_{bias} and backgate voltage V_{BG} in the p-type doping regime, in the region close to the CNP. Bias sweep direction is indicated by the black arrow. Dotted blue and red lines represent respectively the retrapping and switching currents. (**b**) Resistance measured at $I_{bias} = -6\ \mu$A. Maxima in the supercurrent correspond to local minima in the resistance, as highlighted by the black guiding dashed lines. (**c**)

In addition, as discussed for example in Ref. 1, in ballistic devices FP oscillations are observed also at finite magnetic field, showing a B^2 dispersion with respect to the Fermi wavevector k_F. By applying an out-of-plane magnetic field, which results in a Lorentz force to electrons and holes, a shift towards higher charge density of the FP resonances is observed, due to the extra phase accumulation, which depends on the applied field. By considering the phase accumulation in the p-region, the authors in Ref. 1 obtained a semiclassical expression which links the carrier density (and thus the Fermi wavevector k_F), the cavity mode number m and the square of the magnetic field B^2:

$$k_F L = m\pi + \frac{\pi}{2} + \frac{\pi}{6m}(L^2 \frac{e}{h} B)^2 \qquad (1)$$

Dispersion curves corresponding to modes $m = 7 - 13$ are shown in the Landau fan

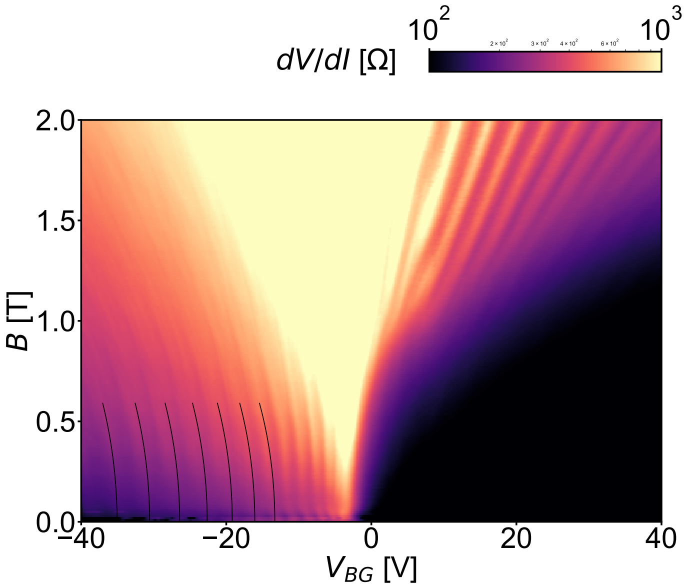

Figure S4: Landau fan diagram as in Fig.2a in main text: the differential resistance dV/dI is measured with a lock-in amplifier by applying a 100 nA AC bias on top of a fixed 200 nA DC bias. Black lines correspond to curves following equation (1) for cavity mode numbers $m = 7 - 13$.

diagram in Fig. S4 and show a very good qualitative agreement with the experimental data.

S2 Additional device characterization

Fig. S5a shows the differential resistance obtained by numerical differentiation of data presented in Fig. 1e of the main text. The supercurrent branch can be identified as the white area at the center of the map corresponding to $dV/dI = 0$.

In a Josephson junction, the differential resistance exhibits sub-gap features originating from multiple Andreev reflections (MARs). MAR features appear at bias voltage values V_b satisfying the condition $V_b = 2\Delta/ne$, up to the point when the voltage drop across the junction is $V_b > 2\Delta/e$ (Δ is the superconducting gap and n an integer number). They are a common feature in Josephson junctions with highly transparent interfaces (as reported for example in Ref. **?**), including Nb-graphene Josephson junctions.[2]

In Fig. S5a, on the p-type doping side, MARs can be identified as darker stripes corresponding to local maxima in dV/dI. They are not observed at fixed I_{bias}, because the

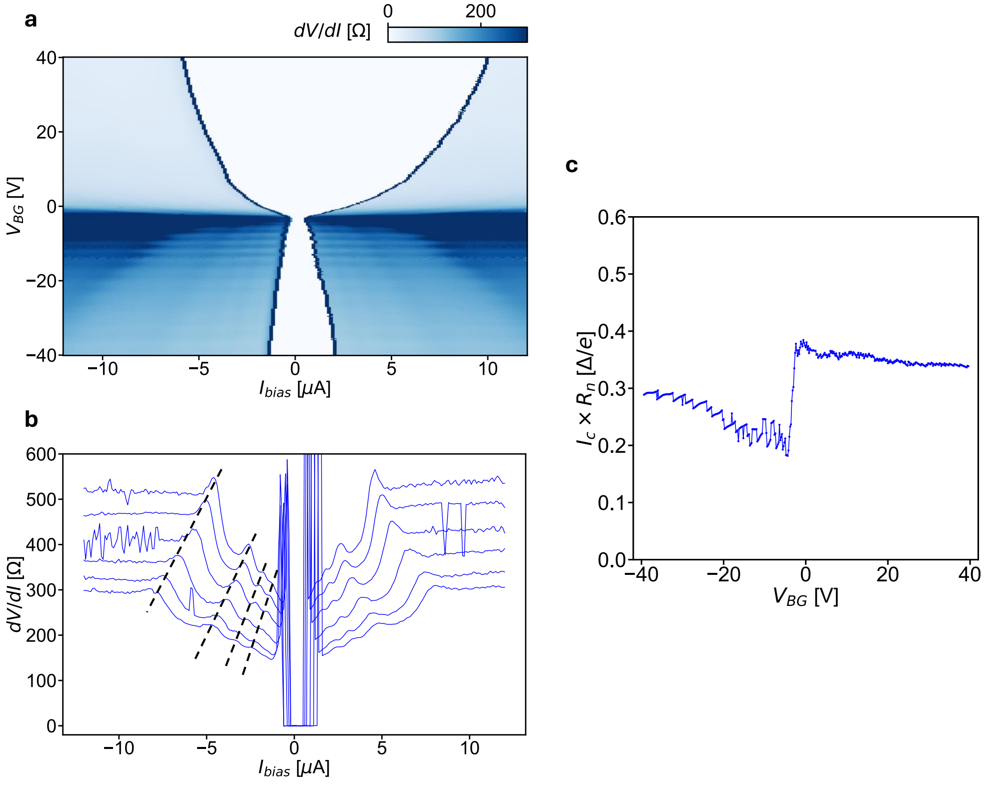

Figure S5: (**a**) Differential resistance dV/dI map as function of DC bias I_{bias} and BG voltage V_{BG}. dV/dI is obtained by numerical differentiation of data shown in Fig. 1e of the main text. (**b**) Selected dV/dI curves corresponding to V_{BG} in a 1 V interval around the CNP point. MAR peaks, here highlighted by the dashed black lines for $n = 1 - 4$, are also visible in the colormap **a**. (**c**) $I_c \times R_n$ product, in units of Δ/e. The critical current I_c is obtained from the supercurrent map shown in Fig. 1e, while the normal state resistance is obtained from Fig. 1c of the main text.

junction's resistance is not constant but modulated by V_{BG}. Selected dV/dI curves as a function of current bias I_{bias} in a 1 V wide range around the CNP are reported in Fig. S5b, where the guiding black dashed lines identify the four visible dV/dI peaks due to MARs (n=$1 - 4$).

A common figure of merit used to assess the quality of a Josephson junction is given by the $I_c \times R_n$ product, which is shown for our device in Fig. S5c in units of Δ/e. The $I_c \times R_n$ product is almost constant on the n-type doping side, while it is lower on the p-type doping side due to the additional p-n interfaces. The authors of Ref. 2 observe that the experimentally obtained value of the $I_c \times R_n$ product is smaller than the theoretical value

equal to $\alpha\Delta/e$, where Δ is the superconducting gap and $\alpha = 2.1$. The $I_c \times R_n$ for our device corresponds to $\alpha \leq 0.4$, comparable to the value obtained in Ref. 2 for a 400 nm junction with Nb-contacts.

S3 Flux focusing

As reported in the main text, a focusing factor $\simeq 1.7$ is obtained from the position of the first minima of the Fraunhofer pattern. This is compatible with what we would obtain by estimating the focusing area of the Nb leads (i.e., the area of the leads that expels magnetic field inside the graphene channel). Following the argument reported in Ref. 3, this can be approximated as the area of the Nb leads that is closer to the graphene channel than every other edge. For our junction's geometry, this corresponds to the trapezoidal shape shown in Fig. S6 in dark grey. Given the Nb leads dimensions ($L = 400$ nm, $W = 3$ μm), the focusing area for each Nb lead is $A_{foc} = 0.55$ μm^2, thus the total focusing area $2A_{foc} = 1.1$ μm^2. This is roughly 90% of the area of the graphene channel, from which we estimate a theoretical focusing factor $\simeq 1.8$, compatible with the value $\simeq 1.7$ extracted from first minima position.

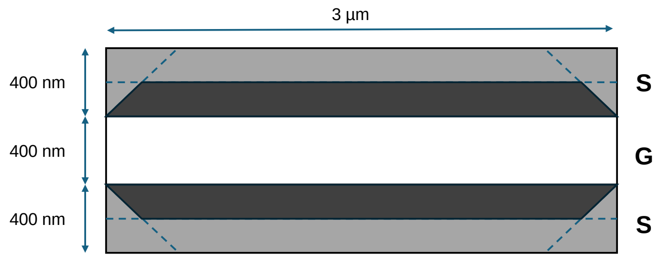

Figure S6: Junction's geometry: the focusing area is calculated as the trapezoidal shape indicated in dark grey in the Nb leads, which corresponds to the Nb lead region which is closer to the Nb-graphene edge than any other edge. S = superconductor. G = graphene.

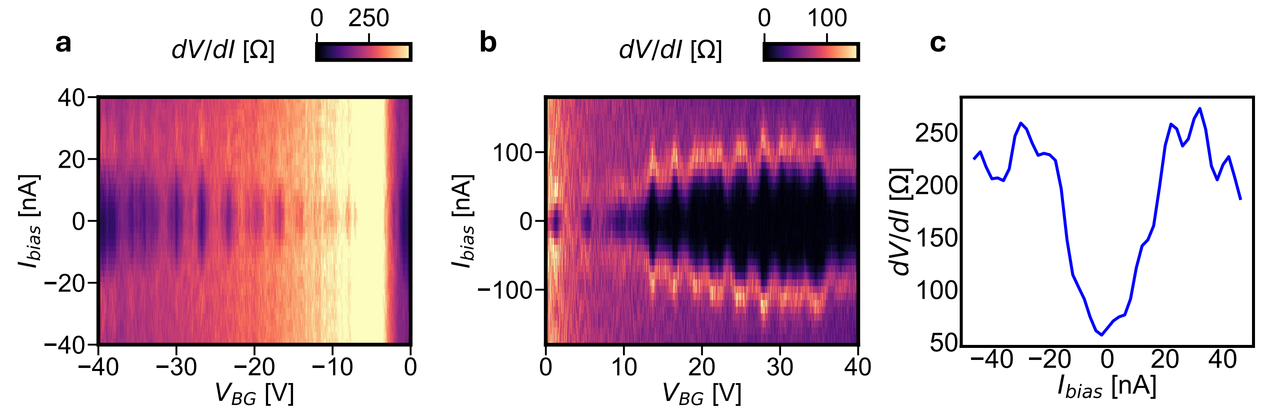

Figure S7: (**a**,**b**) Differential resistance dV/dI *versus* BG voltage V_{BG} and DC bias current I_{bias}. Applied AC bias: 1 nA. $B = 200$ mT, $T = 240$ mK. (**c**) Example dV/dI linecut ($V_{BG} = -39.3$ V).

S4 Superconducting pockets in the semiclassical regime

In Fig. S7 we report the observation of superconducting pockets in the semiclassical regime. Figs. S7a,b show the differential resistance dV/dI as a function of V_{BG} and I_{bias} at fixed magnetic field, $B = 200$ mT. Pockets are observed in both p-type and n-type doping regimes (similar evidence was reported in Ref. 3, while no traces of supercurrent for p-type doping were reported in Ref. 2 for junctions with Nb contacts). A linecut example of a pocket in the p-type doping regime ($V_{BG} = -39.3$ V) is shown in panel **c**. Compared to the QH regime, pockets with larger I_c are observed (here up to ~ 100 nA).

In the semiclassical regime, superconducting states have been attributed to Andreev bound states made of closed trajectories formed by the combination of Andreev reflections at the graphene-superconductor interface and random elastic scattering at the graphene-vacuum interface.[2] This "chaotic ballistic billiard", as defined by the authors, leads to the formation of closed loop trajectories which are capable of transferring Cooper pairs between the superconducting contacts, despite electrons and holes moving along different non-retracing paths.

S5 Temperature dependence of the pocket amplitude in the QH regime

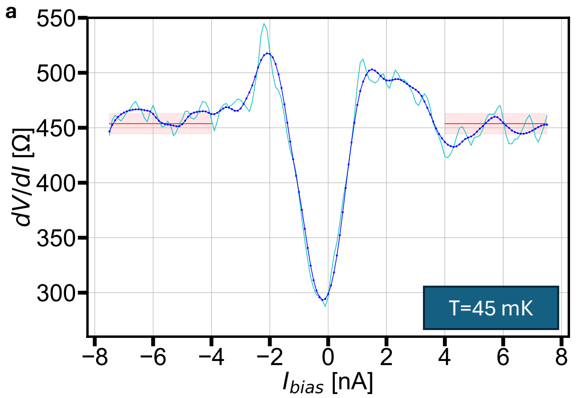

Figure S8: Differential resistance dV/dI *versus* DC bias current I_{bias}. Light blue line is the raw data, the blue line is the smoothed data, as explained in the text. Red shaded areas are used to calculate the normal state resistance. (**a**) Data for $T = 45$ mK. (**b**) Data for $T = 300$ mK.

Here we discuss the temperature dependence of the amplitude of the superconducting pockets in the quantum Hall regime. The investigated pocket is located at $B = 1.6$ T, $V_{BG} = 11.6$ V (same starting point for analysis shown in Fig. 4 of main text). We define the

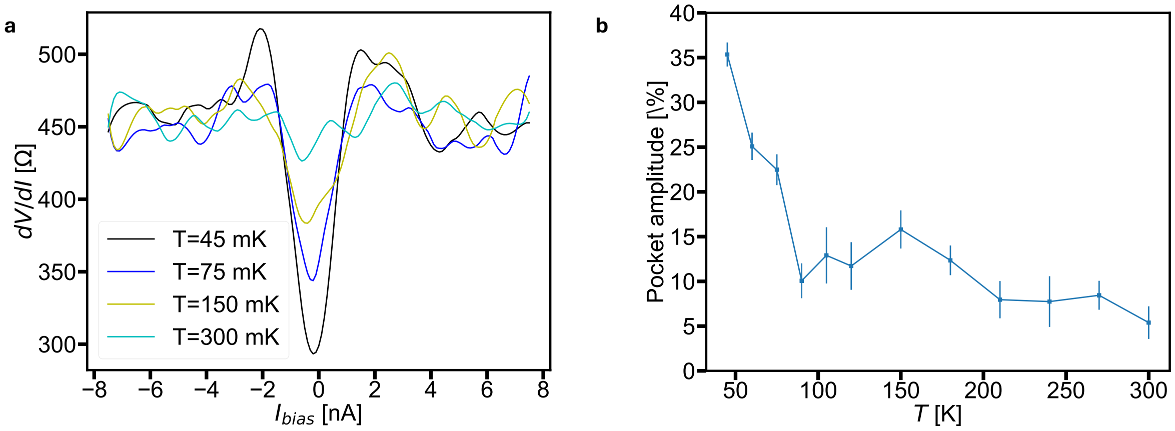

Figure S9: (**a**) dV/dI as a function of the DC bias current I_{bias} for selected temperature values. (**b**) Pocket amplitude as a function of the temperature, calculated as explained in the text. Errorbars are obtained by taking into account the error on the normal state resistance value (estimated as standard deviation, red shaded regions in Fig. S8).

amplitude of a pocket as the ratio between the zero-bias dV/dI minimum and the normal state resistance. Based on this definition, a pocket has amplitude 1 (100%) if $dV/dI = 0$ at 0 bias (total suppression of dV/dI); on the opposite, in the case of dV/dI at 0 bias equal to the normal state resistance value, no superconducting pocket is present (it has 0 amplitude). The normal state resistance value is calculated as the mean value of the measured differential resistance at large enough (positive and negative) DC bias ($I_{bias} > 4$ nA) to suppress the supercurrent. As an example we report in Fig. S8 two acquisitions, at $T = 45$ mK (panel **a**) and $T = 300$ mK (panel **b**). Red lines indicate the calculated mean value of the resistance, while the shaded red areas correspond to the standard deviation of this value.

Prior to calculating the pocket amplitude, a mild smoothing operation was performed (implemented through the `savgol_filter` function of `scipy.signal` Python package,[4] by setting $W = 26$ and $n = 5$). In Fig. S8 both raw (thin light blue continuous line) and smoothed data (blue dot-line) are reported.

The amplitude of the superconducting pocket is rapidly suppressed with increasing temperature, and becomes negligible ($< 10\%$) for $T > 200$ mK, as can be seen from Fig. S9a. The overall temperature dependence is shown in Fig. S9b. As discussed in Ref. 3, the Josephson energy $E_J = \hbar I_c/2e$, which is of the order of 2 μeV for supercurrents of 1 nA, is of the same order of magnitude and very close to the thermal energy scale $k_B T \simeq 2.6$ μeV at $T = 30$ mK. By increasing the temperature, the thermal energy rapidly exceeds the Josephson energy, thus destroying the supercurrent in the QH regime.

S6 Coupling of B-modulated supercurrents and sum frequency in the FFT spectrum

In Fig. 4c of the main text, we show that a fixed-ν acquisition reveals two main periodicities of the supercurrent pockets, indicating interference in the whole junction area and in the smaller area defined by doping. Additionally, a third peak in the FFT spectrum appears at

a frequency that quantitatively corresponds to the sum of the two main frequency components. The observation of this third peak provides evidence of the coupling of two B-field-modulations of the supercurrent in the two different areas, which we explain in detail in the following.

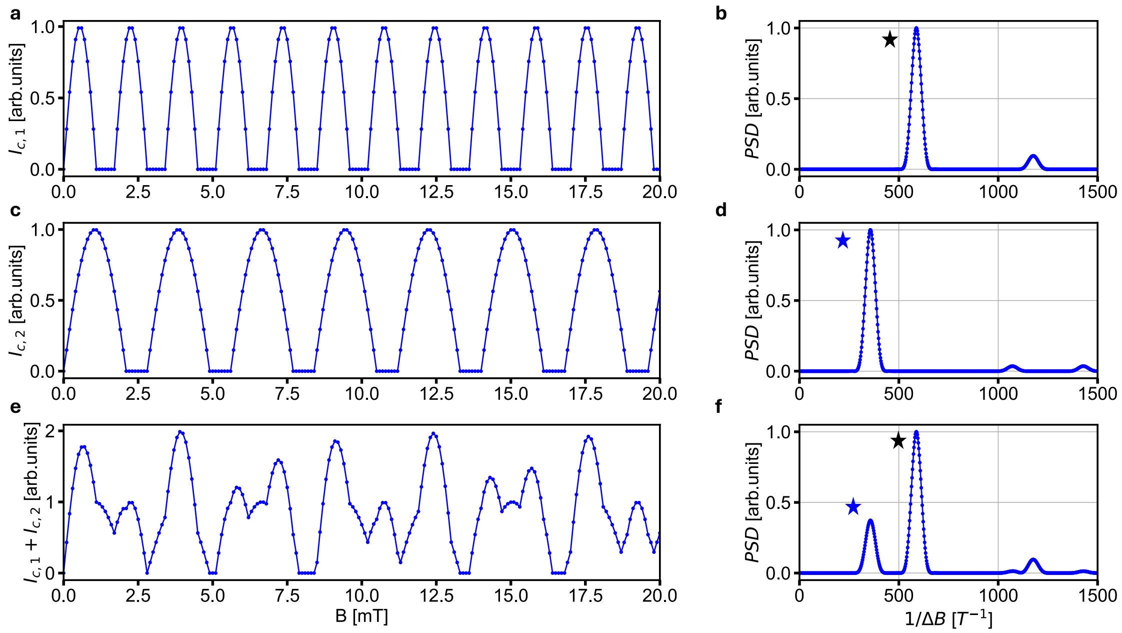

Figure S10: Simulation of independent B-modulated supercurrent pockets. (**a**) $I_{c,1}$ vs. B. (**b**) Fourier transform (power spectral density) of the data in panel **a**. The peak indicated with the black star corresponds to the periodicity of the simulated pockets in **a**. (**c**) $I_{c,2}$ vs. B. (**d**) Fourier transform (power spectral density) of the data in panel **c**. The peak indicated with the blue star corresponds to the periodicity of simulated pockets in **c**. (**e**) $I_{c,1} + I_{c,2}$ vs. B. (**f**) Fourier transform (power spectral density) of the data in panel **e**. The peaks indicated with the black and blue stars correspond to the individual periodicities of $I_{c,1}$ and $I_{c,2}$, but the third peak at the sum frequency is absent.

If the two modulations were independent, we would find only two peaks in the FFT spectrum. This case is shown in Fig. S10, where we simulate two B-modulated critical currents ($I_{c,1}$, Fig. S10a and $I_{c,2}$, Fig. S10c) and their sum ($I_{c,1}+I_{c,2}$, Fig. S10e). We consider discrete pockets (i.e., with extended intervals of suppressed supercurrent) as observed experimentally. The non-sinusoidal shape of $I_{c,1}$ and $I_{c,2}$ gives rise to overtones in the FFTs (Figs. S10b,d), but the third peak at the sum frequency is absent in Fig. S10f.

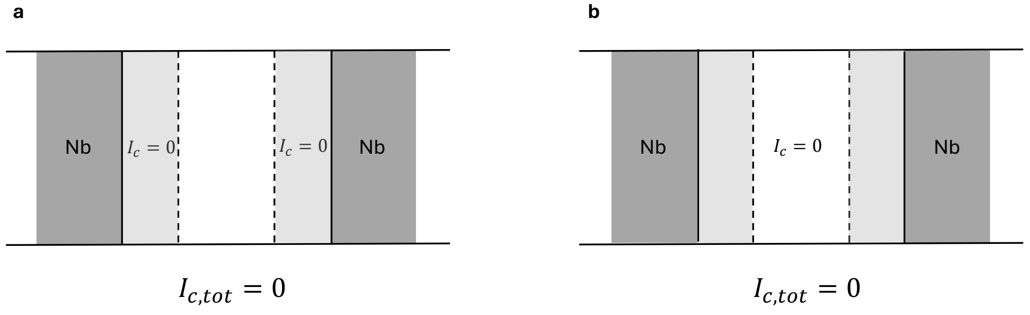

Figure S11: (**a**) Schematics of the junction when destructive interference takes place in the regions close to Nb contacts. (**b**) Schematics of the junction when destructive interference takes place in the cavity originated by the doping variation. In both cases, the total supercurrent through the junction is equal to 0.

In our device, however, the two modulations are not independent. Specifically, if destructive interference takes place in the regions close to the leads, hence the critical current there is 0 (as schematically shown in Fig. S11a), no supercurrent can flow through the junction. Analogously, destructive interference in the cavity (Fig. S11b) prevents any supercurrent flow across the device. Indeed, destructive interference over one area necessarily suppresses the supercurrent of the whole junction. In conclusion, when the two B-modulations are not independent, the smallest critical current dominates (which also includes the condition that, if any of the two supercurrents is 0, then the total supercurrent is also 0).

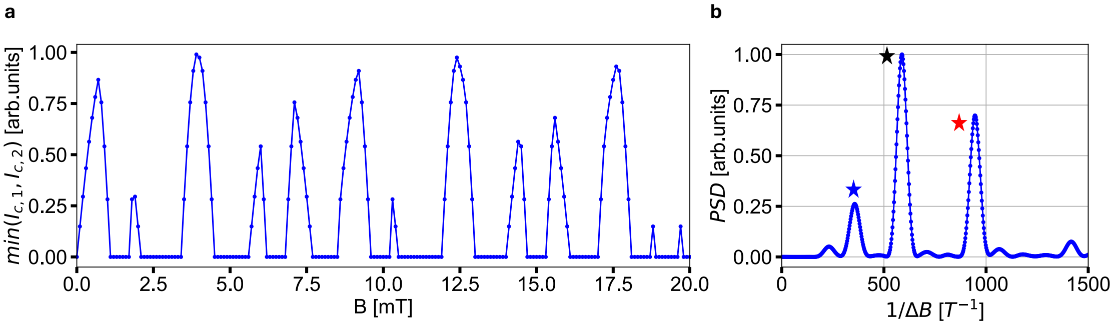

Figure S12: (**a**) Minimum between $I_{c,1}$ and $I_{c,2}$ as a function of B. (**b**) Fourier transform (power spectral density) of data in panel **a**. Peaks labelled with a black and a blue star correspond respectively to the periodicities of $I_{c,1}$ and $I_{c,2}$, as shown in Fig. S10. The third peak, labelled with a red star, is located at a frequency corresponding to the sum of the two main oscillatory components.

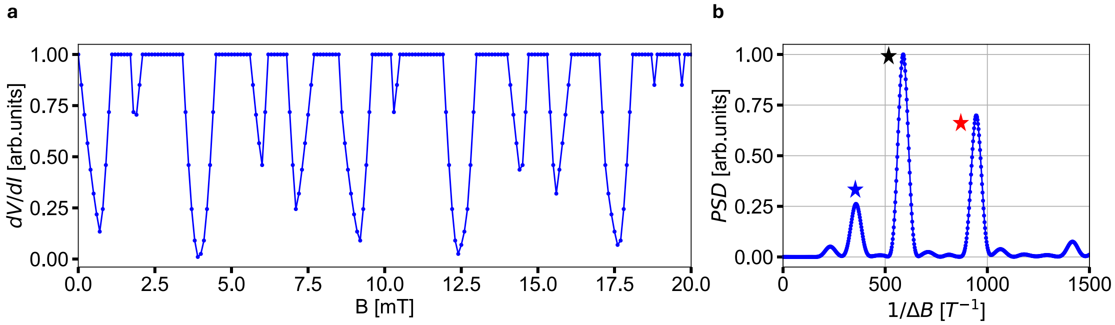

Figure S13: (**a**) Simulation of dV/dI from the critical current shown in Fig. S12a. The differential resistance is suppressed when the critical current is different from zero. (**a**) Fourier transform (power spectral density) of data in panel **a**. The FFT spectrum corresponds to the one in Fig. S12b: the third peak is also present.

The minimum between $I_{c,1}$ and $I_{c,2}$ and the corresponding FFT spectrum are reported in Fig. S12. The FFT clearly shows a third peak (labelled with a red star), located at the sum frequency of the two fundamental modes.

In the experiment, we rely on the measurement of zero-bias dV/dI, which is modulated in the same way as the critical current (dV/dI is suppressed when $I_c \neq 0$). In Fig. S13 we show a simulation of dV/dI and the corresponding FFT, again showing the three peaks.

References

(1) Calado, V. E.; Goswami, S.; Nanda, G.; Diez, M.; Akhmerov, A. R.; Watanabe, K.; Taniguchi, T.; Klapwijk, T. M.; Vandersypen, L. M. K. Ballistic Josephson junctions in edge-contacted graphene. *Nature Nanotechnology* **2015**, *10*, 761–764.

(2) Ben Shalom, M.; Zhu, M. J.; Fal'ko, V. I.; Mishchenko, A.; Kretinin, A. V.; Novoselov, K. S.; Woods, C. R.; Watanabe, K.; Taniguchi, T.; Geim, A. K.; Prance, J. R. Quantum oscillations of the critical current and high-field superconducting proximity in ballistic graphene. *Nature Physics* **2016**, *12*, 318–322.

(3) Amet, F.; Ke, C. T.; Borzenets, I. V.; Wang, J.; Watanabe, K.; Taniguchi, T.;

Deacon, R. S.; Yamamoto, M.; Bomze, Y.; Tarucha, S.; Finkelstein, G. Supercurrent in the quantum Hall regime. *Science* **2016**, *352*, 966–969.

(4) Savitzky, A.; Golay, M. J. E. Smoothing and Differentiation of Data by Simplified Least Squares Procedures. *Analytical Chemistry* **1964**, *36*, 1627–1639.
